# Without Decorations at Grimsby

**Published:** 1914-01-03

**Authors:** 


### Without Decorations at Grimsby.

CHRISTMAS at this hospital begins with a tea and concert for the adult patients and their friends. The concert is a very good one, and is usually got up by outside friends. The men are allowed to smoke, which, of course, is a great joy to them.

Christmas morning all the patients receive a parcel of clothing, Christmas letters, etc., and the children have a stocking filled with toys. The wards are made bright with plants and flowers (other decorations not being allowed now). New jackets (pink for women and children, red for men), ribbons, pinafores, etc., are worn and the patients ready for the committee by 12 o'clock. Dinners, which consist of turkey, plum-pudding, jellies, etc., are then served. The Mayor carves in the first ward, the Chairman in the second, and an ex-Chairman in the third. After dinner the men have a smoke, and then all patients must rest until the "Service" at 4 o'clock. Tea is at 5 o'clock, and during the evening half-hour concerts are held in the wards. The patients are then got ready for the night and wards closed about 8.30 P.M., after a very enjoyable day.

Boxing Day is the children's day. The Christmas-tree, which has been a source of wonder to them since Christmas Eve, is going to be stripped by Father Christmas at 4 o'clock. All who can be moved go to the children's ward, and everyone in the hospital gets a present. Intense excitement prevails, but it is soon over, and the children go to sleep hugging their dolls.

The nurses get a lot of enjoyment out of all the extra hard work, but their special treat is a fancy-dress dance, with a sit-down supper. Plenty of partners are invited, and private nurses are engaged to look after the patients for the evening, so all have a thoroughly good time.

On New Year's Eve the nurses give a competition tea to the rest of the staff and a few outside friends. Hampers from home provide the cakes, etc. Two senior nurses arrange the tables and act as hostesses. Being responsible for this afternoon gives the nurses a lot of pleasure and makes them feel they are taking an active part in the Christmas arrangements.

Maids, like nurses, get more work during Christmas, but arrangements are made for them to get some time at the various concerts, etc. They have their dinner on Boxing Day at 2.30 P.M., the matron and sisters waiting on them. Then a present from the Christmas-tree, and after all teas are over and supper tables laid they are given late leave and a ticket for the theatre. One night during Christmas week they dance for about two hours. The main corridor only is decorated, a different style being adopted each year. Ward decorations were discontinued four years ago, as they were the root of a lot of trouble and no real good to the patients. General present-giving is not allowed. Outside collections are made by two friends of the hospital for ward plants and flowers, ribbons, etc., and the sisters are allowed to put a box out each visiting day for any money that the patients' friends can spare. The committee also make a reasonable allowance for Christmas out of the funds.

